# Determination of efficacy and toxicity of diclofenac microemulsion formulation for musculoskeletal pain: an observational study

**DOI:** 10.1186/s13104-020-05120-3

**Published:** 2020-06-12

**Authors:** Hoan Linh Banh, Andrew Cave

**Affiliations:** Department of Family Medicine/Faculty of Medicine and Dentistry, University of Alberta, 6-10 University Terrace, Edmonton, AB T6G 2T4 Canada

**Keywords:** Microemulsion, Transdermal formulation, Diclofenac, Pain, Topical foam

## Abstract

**Objective:**

Musculoskeletal pain is often caused by injury to the bones, muscles, tendons, ligaments or nerves. Symptoms can be localized or generalized. Mild-moderate symptoms are treated with topical/oral over the counter drugs. Microemulsion delivery formulations are thermodynamically stable, have superior bioavailability and better penetration of lipophilic and hydrophilic drug into the dermis. A prospective observational study in patients: 18 years or older, with mild-moderate musculoskeletal pain; with severe pain without adequate pain control; with severe pain and could not tolerate oral agents; with renal impairment were invited to try diclofenac 2% in microemulsion foam. They were followed up at 2 and 4 weeks. A 50% reduction on a visual analog pain scale was considered success. Adverse events were defined as irritation, gastrointestinal upset/bleed, rectal bleed, and hematemesis. The objective was to determine the efficacy and toxicity of diclofenac 2% in microemulsion foam.

**Results:**

Thirteen consecutive patients with musculoskeletal pain consented to participate. Two patients were lost to follow up. Two of the 11 patients reported minimal improvement, while nine patients reported minimum 50% reduction. No adverse effects were reported. Diclofenac 2% in microemulsion foam is effective in the treatment of mild to moderate musculoskeletal pain and well tolerated.

## Introduction

Musculoskeletal pain is often caused by an injury to the bones, muscles, tendons, ligaments or nerves. The symptoms can be localized or generalized. The mild to moderate symptoms are usually treated with acetaminophen, or non-steroidal anti-inflammatory drugs (NSAID) such as ibuprofen [1]. Currently, several topical formulations of NSAIDs are prescribed for mild to moderate osteoarthritis [2, 3]. The commonly available strength in Canada is a soft, homogenous, cream-like oil-in-water topical emulsion, which contains either diclofenac diethylamine 1.5% w/w (Pennsaid^®^) or diclofenac sodium 1.16% and 2.32% (Emulgel^®^). It is recommended that a total dose should not exceed 320 mg of diclofenac per day [4].

Compounding pharmacies can provide various prescription strengths of diclofenac in pluronic lecithin organogel (PLO). The most common strengths prescribed are 5–10% in PLO gel to be applied once daily or twice daily. Because the PLO gel is poorly absorbed, a higher dose is required to relieve pain. However, topical NSAID is not without side effects. GI bleed has been observed in clinical trials using the 1.5% diclofenac (Pennsaid^®^) [5]. Also, NSAIDs increase the chance of a myocardial infarction or ischemic stroke that can lead to death [5]. The adverse effects are associated with dose and duration of use of NSAID and in people with a history of heart disease [6].

Concerns were raised by many clinicians about the use of high concentrations of topical diclofenac being used. We explored the possibility of developing a better delivery compound that would require less active drug and provide better or equivalent efficacy. As a result, a microemulsion compound (foam) was developed as a delivery system. In vitro, it has been shown to increase absorption through the dermis when compared to the currently available products [7]. This would allow clinicians to use a lower concentration of diclofenac to achieve the same or increased effects. We describe a case series of 11 patients treated with diclofenac 2% in microemulsion foam for mild to moderate musculoskeletal pain.

## Main text

This is a prospective observational study at a family medicine clinic. All consecutive eligible consenting patients with mild to moderate musculoskeletal pain of a variety of durations were treated with topical 2% diclofenac in microemulsion foam applied as a topical spray. The Northern Alberta Academic Family Physician Fund, a fund intended to support initiatives promoting the advancement of family medicine, provided 3000 Canadian dollars to purchase the active ingredients for the study. The study aimed to recruit 15 patients. The eligibility criteria were as follows:

1. Patients 18 years or older, with mild to moderate musculoskeletal pain. Mild to moderate pain was defined as a patient reported pain with a visual analog pain score ≤ 6 out of 10
2. Patients with severe pain who, despite using other treatment agents such as opioid, acetaminophen or cortisone injection, who still did not receive adequate pain control; or
3. Patients with severe pain who could not tolerate oral NSAID or;
4. Patients with renal impairment in whom oral NSAID use is contraindicated

Exclusion criteria include:

1. Patients who have hypersensitivity to diclofenac; or
2. Patients with open wounds or infection at the application site; or
3. Patients under 18 years of age; or
4. Women who are pregnant; or
5. Patients with more than 1 active painful site.

All patients were provided with two 50 mL bottles of 2% diclofenac in microemulsion foam to be applied twice a day and allowing up to seven applications for each site a day. Each 50 mL bottle contained 1 g of diclofenac in microemulsion solution. Each application contained about 1 mL which is enough in each bottle for 50 applications in the 1-week Patients were followed up in 2 weeks. Pain control was assessed at the follow up visit. If there was patient reported improvement on the VAS, another two bottles were provided and the patients were followed up again in 2 weeks. Improvement is defined as ≥ 50% reduction on visual analog pain scale after 2 weeks. Adverse events were defined as irritation at the application site, gastrointestinal (GI) upset, GI bleed, rectal bleed, vomiting blood, and rash. Verbal comments from the patients about the experience of using the 2% diclofenac in microemulsion foam were also collected and documented at each visit.

The observational study received Research and Ethics approval (Pro00079011) from the University of Alberta. Patients with musculoskeletal pain were identified during their regular visits to the family physician. Patients who needed a change in therapy and met the inclusion criteria were offered the new preparation. They were informed by their clinician about our wish to monitor the effects of this standard medication offered in a new format and written informed consent was obtained from eligible patients for monitoring of the effects. In the first 2 weeks, 13 patients met the eligibility criteria and provided signed consent. Two patients were lost to follow up in 2 weeks. Of the remaining 11 remaining patients, nine patients noted significant improvement in pain control. Some of them requested prescriptions for the 2% diclofenac in microemulsion foam for their family members who were also suffering from musculoskeletal pain. Since, at that time, there was a limited supply of the 2% diclofenac in microemulsion foam for the study, the investigators decided to discontinue enrolling patients. The study patients were given two more bottles of the 2% diclofenac in microemulsion foam for the following 2 weeks. The treatment was extended to use by other patients and physicians by prescription since all constituents are approved therapeutically. At this time other family physicians could provide a prescription to their patients.

## Results

Table 1 summarizes patient characteristics and their treatment responses. A total of 11 patients of 13 completed the study. There were six females. Two of the 11 patients reported minimal improvement. The other nine patients reported at least 50% reduction on the pain scale after the first 2 weeks. During both of 2-week follow up, no patient dropped out due to adverse effects or lack of efficacy. All of the patients reported using no more than four pumps a day. Each pump is equivalent to 1 mL of the 2% diclofenac in microemulsion solution. The remaining volume in the bottles were assessed to ensure that patients were not exceeding seven pumps a day.

**Table 1:** Patient demographics and their treatment response

Age (range)	Patient	Site	Initial pain score (10)	Follow-up pain score (10)	Comment
46–55	1	Both hips	8	4	Concurrent treatment with morphine without relief
56–65	2	Left knee	10	3	Tried diclofenac 10% in PLO gel without relief
	3	Bilateral elbows	7	3	Concurrent treatment with oral ketoprofen
	4	Posterior Cervical Neck	5	2	Works very quickly. Not messy or greasy
	5	Left hip	8–9	7	Has severe back pain also. Received cortisone injections
	6	Left knee	6	2 at rest, 4 during activity	Used diclofenac 1.5% Pennsaid^®^ and 10% in PLO gel in past without relief
	7	Shoulder	6–7	4–5	Received occasional morphine and used diclofenac 10% in PLO without much relief
66–75	8	Right hip	6–7	3	Was using diclofenac 10% in PLO gel but it was too messy. Prefer the new formulation which absorbs very quickly
76–85	9	Right hip	4	0	
	10	Left shoulder	6–7	0–1	Works very quickly not messy
86–95	11	Left foot	9–10	5–6	Used diclofenac 1.5% Pennsaid^®^ without relief

Topical NSAIDs are used as an alternative to oral NSAID for mild to moderate musculoskeletal pain to provide localized drug delivery with equal efficacy but minimizing systemic adverse effects. The maximum recommended daily dose should not exceed 320 mg of diclofenac per site [5]. For lower extremities, the daily dose should not exceed 160 mg of diclofenac to each affected joint and for upper extremities, the maximum daily dose is 80 mg of diclofenac [4]. Table 2 summarizes estimated absorption in milligram (mg) for each concentration used topically.

**Table 2:** Percent of diclofenac absorption [5]

Diclofenac formulation (%)	Diclofenac (mg) after single application (2 g gel)	Diclofenac (mg) after single application (4 g gel)	Total diclofenac absorbed (mg) if daily maximum 32 g of gel used	Absorption in (mg) using 4 g gel 4 times a day in lower extremities	Absorption in (mg) using 2 g gel 4 times a day in upper extremities
1	20	40	320	160	80
3	60	120	960	480	240
5	100	200	1600	800	400
10	200	400	3200	1600	800

In Canada, pharmacies are permitted to provide non-commercially available concentrations of diclofenac in any semisolid base. The common prescription concentrations are compounded in PLO as semisolid base. There is limited published information about the efficacy or toxicity of diclofenac in PLO gel [7, 8]. One study compared diclofenac 2% in PLO gel with placebo for mild to moderate osteoarthritis of the knee [8]. The study, however, did not use a standardized pain assessment score to evaluate the pain response. The authors concluded that diclofenac in PLO gel provided therapeutic values in treating mild to moderate osteoarthritis of the knee.

Microemulsion transdermal drug delivery formulations are thermodynamically stable, and have superior bioavailability and better penetration of lipophilic and hydrophilic drug into the dermis [9, 10]. A study in healthy volunteers showed that microemulsion was tolerated well [11]. In an in vitro study, it was shown that the diclofenac in PLO gel or currently commercially available products released about 50% less of the active drugs compared to the microemulsion formulation (40% vs 80%) [12]. In this study, 2% diclofenac in microemulsion foam provided adequate pain control for the patients. Each 50 ml bottle has a total of 1 g diclofenac. Each 1 ml pump of the diclofenac 2% in microemulsion provides 20 mg of diclofenac. At maximum dose of seven pumps a day, the total daily diclofenac is 140 mg for each site which was substantially below the 320 mg a day recommendation.

This is the first observational study to assess the clinical effects and adverse events using diclofenac powder in microemulsion in the treatment of musculoskeletal pain. Due to the small sample size, the before and after pain intensity statistical tests could not be used to provide meaningful results. Prior to enrolling in the study, five of the 11 patients had used either diclofenac 10% in PLO gel or diclofenac 1.5% or 2.32% alcohol based or Emulgel^®^ without adequate relief. All of these patients, however, reported at least 50% reduction in the visual analogue score from baseline after using diclofenac 2% in microemulsion foam. Patient number 5 reported no response to the diclofenac microemulsion foam. His pain severity could be associated with the patient’s severe back pain which was not treated. Patients number 1 and 11 with severe pain were given the topical formulation because they both have renal insufficiency and they would be at risk of renal function deterioration if given oral NSAID. Patient number 2 was awaiting knee replacement surgery and preferred not to take oral NSAID.

Most notable comments from the patients were that the microemulsion foam is less messy than PLO gel and the absorption was almost instantaneous which resulted in quicker onset of action. No adverse event was reported by the patients in the study.

Diclofenac 2% in microemulsion foam appears to be effective in the treatment of mild to moderate musculoskeletal pain either as a single agent or an adjuvant agent. It was well tolerated.

## Limitations

This is an observational study and the sample size is small. Although a majority of patients had positive response, a higher strength was trialed on the two patients who reported inadequate response.

## Data Availability

This study has no additional supporting data to share.

## Abbreviations

NSAID: Non-steroidal anti-inflammatory drugs
PLO: Pluronic lecithin organogel
GI: Gastrointestinal
mg: Milligram
